# Z2-*γ*: An Application of Zienkiewicz-Zhu Error Estimator to Brain Tumor Detection in MR Images

**DOI:** 10.3390/jimaging8110301

**Published:** 2022-11-05

**Authors:** Antonella Falini

**Affiliations:** Computer Science Department, University of Bari Aldo Moro, 70125 Bari, Italy; antonella.falini@uniba.it

**Keywords:** brain tumor detection, finite-elements, adaptivity, morphological transformation

## Abstract

Brain tumors are abnormal cell growth in the brain tissues that can be cancerous or not. In any case, they could be a very aggressive disease that should be detected as early as possible. Usually, magnetic resonance imaging (MRI) is the main tool commonly adopted by neurologists and radiologists to identify and classify any possible anomalies present in the brain anatomy. In the present work, an automatic unsupervised method called Z2-γ, based on the use of adaptive finite-elements and suitable pre-processing and post-processing techniques, is introduced. The adaptive process, driven by a Zienkiewicz-Zhu type error estimator (Z2), is carried out on isotropic triangulations, while the given input images are pre-processed via nonlinear transformations (γ corrections) to enhance the ability of the error estimator to detect any relevant anomaly. The proposed methodology is able to automatically classify whether a given MR image represents a healthy or a diseased brain and, in this latter case, is able to locate the tumor area, which can be easily delineated by removing any redundancy with post-processing techniques based on morphological transformations. The method is tested on a freely available dataset achieving 0.846 of accuracy and $F_{1}$ score equal to 0.88.

## 1. Introduction

The term brain tumor refers to an abnormal growth of cells in the brain. It could be cancerous or not and could originate directly from the brain tissues or have a metastatic nature. Benign brain tumors have a uniform structure and do not contain active cells; on the contrary, malignant brain tumors have a non-uniform structure and contain active (cancerous) cells. All the types of brain tumors may produce symptoms that vary according to the parts of the brain involved. Moreover, brain tumors may vary in size and location, besides presenting a lot of abnormalities which make it difficult to characterize and identify them.

Magnetic Resonance Imaging (MRI) is a very common tool used in neurology for visualizing the brain anatomy along three different planes: axial, coronal, and sagittal. When protons are placed in a magnetic field, they align. This alignment (magnetization) is then perturbed by introducing an external radio frequency energy. The protons start oscillating and, at this state, they can absorb energy and hence the nuclei can release it or re-radiate it. The nuclei then return to their original equilibrium state: the time that it takes for the relaxation of the component parallel to the magnetic field to go back to the equilibrium is called longitudinal relaxation time or T1; on the other hand, the transversal relaxation time is abbreviated with T2. The transmitted signals are measured; then, the Fourier transform is used to convert the frequency information to corresponding gray scale intensity values: this makes it possible to construct an image. By varying the sequence of the radio frequency pulses applied and collected, different types of images can be created. In particular, calling repetition time (RT) the time interval between successive pulse sequences applied to the same slice, and calling time to echo (RE) the time interval between the delivery of the pulse and the receiving of the echo signal, the so called T1-weighted and T2-weighted images can be created. Images T1-weighted are produced using short TE and TR, while images T2-weighted are produced using longer TE and TR.

The objective of this paper is to describe an automatic methodology to identify any possible brain tumor given either a T1-weighted or a T2-weighted MRI. By screening the given image, the proposed approach locates the tumor area as well as identifies those scans that do not present any abnormal tissues, and hence, can be categorized as healthy brains. The method described here relies on the use of a finite-elements approximation of the input image and on the use of the Zienkiewicz-Zhu (Z2) error estimator [1,2,3] to adaptively and locally refine the resolution of the image at specific sites. In particular, any area of strong change in the gray intensity values will be easily identified by the Z2 error estimator, allowing for highlighting the abnormal tissues.

While the use of finite-elements and adaptive strategies is not new in the image segmentation context, see [4,5,6,7,8], these methods are rather complex as they are framed in a variational formulation, and their objective is different from the one proposed here: the given image is split into several parts or components that can be easily identified via the location of edges. Hence, these methodologies aim at locally reconstructing the profiles of these regions, but they do not address the problem of the classification of such regions.

In general, regarding the brain tumor detection task, many methods are developed by using statistical and machine learning techniques; see [9,10]. In particular, advanced architectures like convolutional neural networks (NN) [11], probabilistic NN [12,13], deep NN [14], and U-Nets [15] have been successfully applied to the task of image segmentation and brain tumor classification; see also [16] for a comprehensive review. On the other hand, many unsupervised methods exist as well; some are based on clustering techniques [17,18,19,20,21,22]; others are based on automatic thresholding methods and morphological transformations [23,24,25,26,27]. Some techniques apply a suitable projection in lower dimensional spaces in order to get rid of unwanted redundancies, and so classical matrix decompositions like the SVD or the non-negative matrix factorization are applied; see [28]. A histogram-based gravitational optimization algorithm [29], Gabor-wavelets [30], and other big data analytics techniques are also employed; see the review [31] and references therein.

Many other approaches are based on supervised anomaly detection: the main goal is to identify abnormalities, given only normal data in the training set, see [32,33,34,35,36,37]. The method introduced here can be interpreted as an unsupervised anomaly detection technique. Besides using the Z2 error estimator, it also exploits specific γ corrections which help to increase or to reduce brightness of the normal tissues surrounding the tumor, according to which modality of MRI is analyzed, i.e., either T1-weighted or T2-weighted. As a final step, the use of morphological operators allows to get rid of redundant noise and to locate the affected, i.e., anomalous, areas inside the brain tissues.

Recent approaches have also exploited particle swarm optimization [38], genetic and evolutionary algorithms; see [39] and references therein. The main contributions proposed in this paper can be summarized as follows:

- A suitable γ correction is applied to pre-process the data;
- The use of finite-elements and Z2 error estimator with isotropic mesh refinement allows for automatically detecting the anomalous areas, if present;
- A post-processing phase, based on the use of morphological transformations, allows for getting rid of the cortex and better locating the anomalous tissues, if present.

The paper is organized as follows: In Section 2, the finite-elements mathematical framework is described. In Section 3, the adopted pre-processing and post-processing are discussed. In Section 4, the method is tested and compared with other available techniques. Section 5 provides some conclusive remarks.

## 2. Mathematical Framework

**Figure 1:**
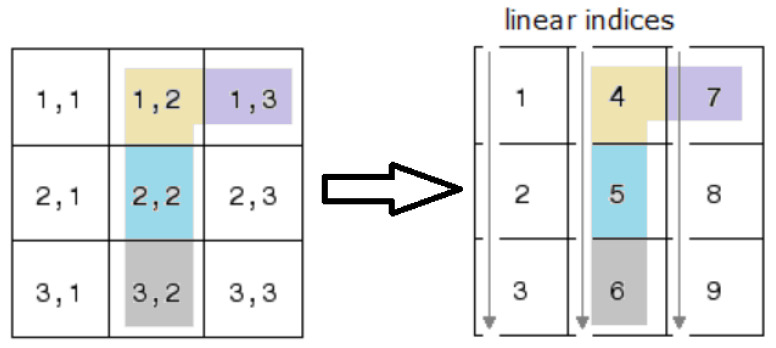
Mapping from classical two subscripts notation of matrix elements to global linear indexing.

Providing as input an MR image I having size m×n pixels, the following methodology produces a finite-elements approximation on a locally refined regular triangulation. In particular, a denser number of triangles will be automatically located where the anomalous tissues are present, allowing for an easy detection of such regions. The first step consists of constructing a regular quadrangular grid associated with I, i.e., every pixel is a node in such a grid. Then, every regular square is diagonally divided in two triangles. In this way, a non-overlapping regular triangulation $T_{h}$ can be obtained, where the subscript *h* denotes the maximal diameter of the triangular elements. At this early stage, $h_{T}=diam\left(T\right)=h$ constant for every triangle $T\in T_{h}$. More in general, $diam\left(T\right):=\max_{x,y\in T}∥x-y∥$ and hence $h:=\max_{T\in T_{h}}h_{T}$. On the constructed triangulation, the space of linear finite-elements can be defined:

(1) $$\begin{array}{c} V_{h}^{1}:=\left\{v_{h}\in C^{0}\left(\overset{-}{\Omega }\right):v_{{h|}_{T}}\in P_{1},\forall T\in T_{h}\right\}, \end{array}$$

where Ω:=(0,m)×(0,n); $P_{1}$ is the space of polynomials of a degree less than or equal to 1, and hence $V_{h}^{1}$ denotes continuous piecewise linear polynomial functions on every triangle *T*. Every function $v_{h}\in V_{h}^{1}$ can be expressed as a linear combination of the basis functions $\left\{\varphi_{k}\right\}$ as

(2) $$\begin{array}{c} v_{h}\left(x\right)=\sum_{k=1}^{N_{h}}v_{k}\varphi_{k}\left(x\right), \end{array}$$

with $v_{k}$ values of the function $v_{h}$ at the grid nodes and $N_{h}$ total number of grid nodes in $T_{h}$. In our case, a continuous approximation image *I* is constructed by interpolating the original image I at the provided pixel values, i.e., via using expression (2),

(3) $$\begin{array}{c} I\left(x\right)=\sum_{k=1}^{N_{h}}I_{k}\varphi_{k}\left(x\right), \end{array}$$

where $N_{h}$ denotes the total number of nodes in $T_{h}$, and a linear global index *k* is used to identify every pixel (i,j) in I, according to the mapping shown in Figure 1. In particular, *k* can be obtained as

$$\begin{array}{c} k:=(j-1)m+i. \end{array}$$

As a second step, a downsampling of the constructed approximation *I* in Equation (3) is performed. In particular, *I* is approximated itself via using a coarser triangulation ${\tilde{T}}_{\tilde{h}}$ having ${\tilde{N}}_{\tilde{h}}$ vertices, where the set of these nodes is not necessarily a subset of the original triangulation nodes. This new approximation shall be denoted as ${\tilde{I}}_{\tilde{h}}$. The third step consists of computing the Zienkiewicz-Zhu error estimator $\eta_{\tilde{T}}$ on every triangle $\tilde{T}\in T_{\tilde{h}}$:

(4) $$\begin{array}{c} \eta_{\tilde{T}}:={∥G\left({\tilde{I}}_{\tilde{h}}\right)-\nabla {\tilde{I}}_{\tilde{h}}∥}_{L^{2}\left(\tilde{T}\right)}. \end{array}$$

In our case, the function $G({\tilde{I}}_{\tilde{h}})$ in Equation (4) is constructed by evaluating the gradient $\nabla {\tilde{I}}_{\tilde{h}}$ on the barycenter of each triangle and then by averaging on those triangles which share a common vertex. In particular, the reconstruction $G_{\tilde{k}}$ of the gradient at every node $\tilde{k}$ of the triangulation ${\tilde{T}}_{\tilde{h}}$ is done by

(5) $$\begin{array}{c} G_{\tilde{k}}:=\sum_{\tilde{T}∋\tilde{k}}\frac{|\tilde{T}|}{|T_{\tilde{k}}|}\nabla {\tilde{I}}_{\tilde{h}}{|}_{\tilde{T}}, \end{array}$$

where the symbol |·| in formula (5) denotes the area of the considered element, and $T_{\tilde{k}}$ denotes the union of the triangles which share the vertex $\tilde{k}$. The elements $\tilde{T}$ having a bigger $\eta_{\tilde{T}}$ are the ones where the intensity value of the pixels varies the most. Then, the estimators for every triangle are sorted from the smallest to the biggest one and the triangles that are marked, and hence refined, are the ones such that

(6) $$\begin{array}{c} \eta_{\tilde{T}}\geq c_{ref}\eta_{{\tilde{T}}_{t}}, \end{array}$$

with $t:=pN_{t}$, $N_{t}$ the total number of triangles and *p* percentage of triangles that will be considered, while $0<c_{ref}<1$. The marking strategy is fundamental to guarantee a specific order of convergence for the chosen approximant; see [40,41]. However, this is not the main concern to be addressed for the current application. The parameters setting for Equation (6) in the tests has been experimentally carried out.

**Remark** **1.** *Although more accurate formulas exist for the gradient recovery estimation (see [42,43,44,45]), in this work, the choice of Equation (5) is intentional: a more rough approximation is indeed preferable in this context rather than an extremely accurate one, as the main goal of the proposed methodology is to estimate the region extension of any possible anomalous tissue, rather than just delineating the profile or the edges of such regions.*

The refinement strategy is repeated for several iterations, until the maximum $\eta_{\tilde{T}}$ does not reach the chosen threshold value, producing every time an isotropic triangulation. At the end, smaller triangles will be located in the regions of interest. Therefore, a final binary map *b* is produced by constructing $b\in V_{\tilde{h}}^{0}$ as a piece-wise constant polynomial function, which is defined as

(7) $$\begin{array}{c} b_{|\tilde{T}}:=\left\{\begin{array}{cc} 1 & \mathrm{if}|\tilde{T}|\mathrm{is}\;\mathrm{minimal},\\ 0 & \mathrm{otherwise}. \end{array}\right. \end{array}$$

It should be noticed that having an isotropic triangulation is a fundamental step in order to produce feasible results with the described strategy. Indeed, having an anisotropic triangulation does not necessarily guarantee that the regions of interests are covered with triangles of a small area. The shape of the triangles can be rather elongated and flattened, preventing the use of the rule in Equation (7) to produce a suitable binary map *b*.

### A Metastatic Brain Tumor Example

**Figure 2:**
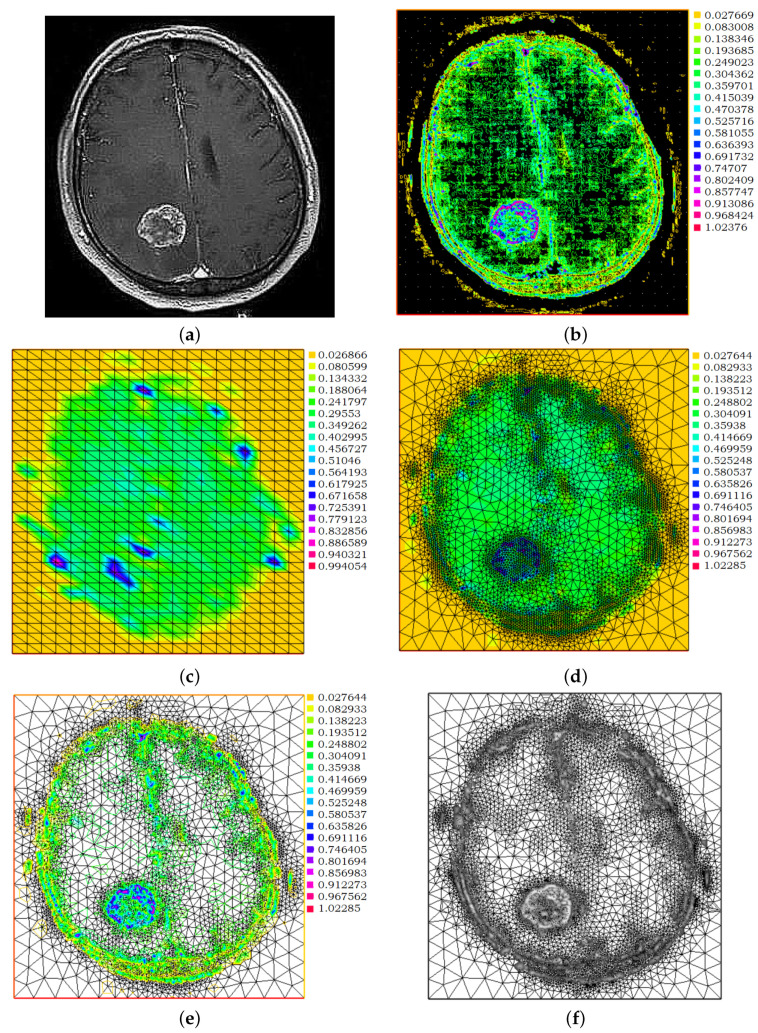

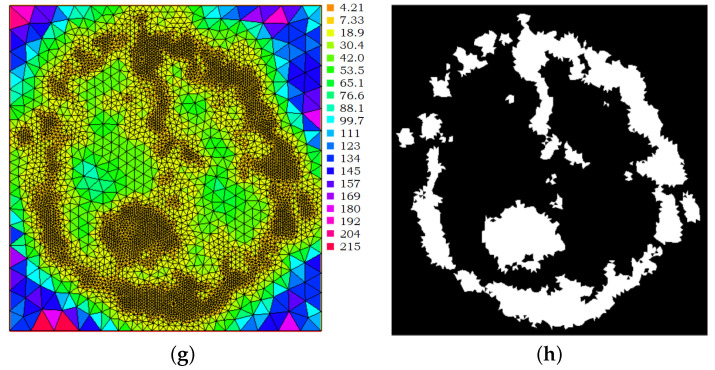
Exemplification of the procedure described in Section 2. Given the input MR scan I(**a**), a linear finite-elements interpolant *I* is constructed (**b**) and successively downsampled to ${\tilde{I}}_{\tilde{h}}$ on a coarser grid (**c**). Adaptive refinement is then performed to get final ${\tilde{I}}_{\tilde{h}}$ on the final ${\tilde{T}}_{\tilde{h}}$ (**d**) resulting in a denser triangulation around the brightest regions as it is shown by the isolines (**e**) and by superimposition (**f**). The triangles of the final ${\tilde{T}}_{\tilde{h}}$ are colored according to their area (**g**). Only the triangles with smaller areas are selected to create a binary map *b* obtained from Equation (7) (**h**).

To make the described procedure clearer to the reader, the following example shows the various employed steps. An image 283×295 pixels of a metastatic brain tumor is selected, Figure 2a. The first interpolant, *I*, obtained with linear finite-elements and defined with 27,828 degrees of freedom (dof), is shown in Figure 2b, while the starting approximation ${\tilde{I}}_{\tilde{h}}$ for the conducted analysis, on a down-sampled triangulation consisting of $N_{t}=1200$ triangles and 217 dof, is shown in Figure 2c. The triangulation is locally refined by using the error estimator in Equation (4) and setting the parameters p=0.25 and $c_{ref}=0.60$ for the Equation (6). The output of this process in shown in Figure 2d,e, where the produced approximant ${\tilde{I}}_{\tilde{h}}$ is defined with 936 dof. In Figure 2f, only for visualization purposes, the original MRI bright regions are superimposed onto the final triangulation in order to show the coherence of the obtained results. Finally, in Figure 2g, the final triangulation is shown again, but the triangles are colored according to their area value. In Figure 2h, the produced binary map *b* is constructed from the final triangulation by selecting only the triangles with a minimal area and setting the corresponding pixels to 1, while setting any other pixel value equal to 0. It is evident how there is still some noise affecting the quality of the produced results. Therefore, this final image will be post-processed as described in Section 3.2.

## 3. Experimental Results

The proposed method refines areas around those pixels where there is a relevant change in the gray intensity values. Therefore, it is prone to increase the resolution of the triangulation near extremely bright regions, or near extremely dark regions when the surrounding pixels have lower and higher intensity values, respectively. Given an MR-T1 or MR-T2 image, bright areas, as well as dark ones, are not necessarily neoplasic tissues. In order to avoid refining unnecessary areas and to give a false prediction for an healthy brain, it is convenient to pre-process the images as follows.

### 3.1. Pre-Processing

**Figure 3:**
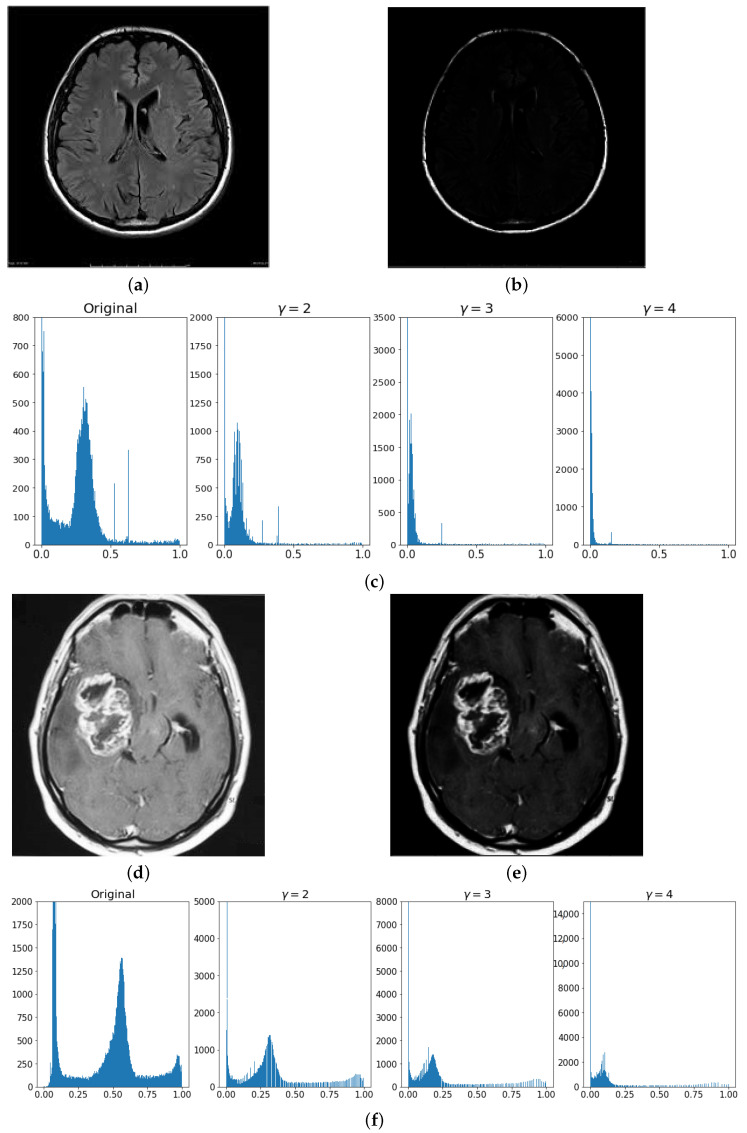
Gamma correction results. (**a**) MR scan I of an healthy brain; (**b**) image G of an healthy brain with γ=4; (**c**) intensity values distributions obtained by varying the parameter γ for an healthy brain MRI; (**d**) affected brain MR I scan; (**e**) affected brain, G image with γ=4; (**f**) intensity value distributions obtained by varying the parameter γ for an affected brain.

Every gray MR acquired image is firstly scaled in the interval [0,1]. Then, in order to reduce the difference in intensity values that could occur around healthy regions that otherwise would be detected as anomalous, a simple gamma correction is applied. In more detail, given I, a gray image scaled in [0,1], the following transformation is applied:

$$\begin{array}{c} G:=I^{\gamma }, \end{array}$$

with γ=4. The aim of the proposed gamma correction is to make the bright pixels even brighter while making the dark pixels even darker. The results can be visually appreciated in Figure 3. In particular, it is evident how, for a healthy brain, Figure 3a, the gamma correction would make any pixel intensity value uniform besides the external cortex; see Figure 3b. More specifically, the intensity values distribution is transformed to obtain a neat separation between the white and black pixels, removing any intermediate shades, Figure 3c. However, for an affected brain, Figure 3d, the neoplasic region would still be enhanced and hence detected, see Figure 3e. In the distribution of the intensity values, there will be evident “steps” (i.e., discontinuities) between any bright and black pixels; see Figure 3f.

**Remark** **2.** *At the moment, the choice for the γ parameter is not automatically done, according to the provided input MR image, but the experiments carried out on the chosen datasets confirmed that γ=4 is the most suitable value. The experimental choice for the parameter γ represents the main limitation of the proposed methodology. Besides standard methodologies, based on histogram equalization, recent approaches exploit the use of meta-heuristic algorithms in order to limit the loss of information [46,47].*

### 3.2. Post-Processing

Once the smaller triangles have been selected and the binary map *b* produced, to better delineate the tumor area and to get rid of the cortex profile, as well as any other noisy area, a morphological transformation called erosion (see Chapter 5 in [48]) is applied with the following kernel:

(8) $$\begin{array}{c} Kernel:=\left(\begin{array}{ccc} 1 & 1 & 1\\ 1 & 1 & 1\\ 1 & 1 & 1 \end{array}\right). \end{array}$$

**Figure 4:**
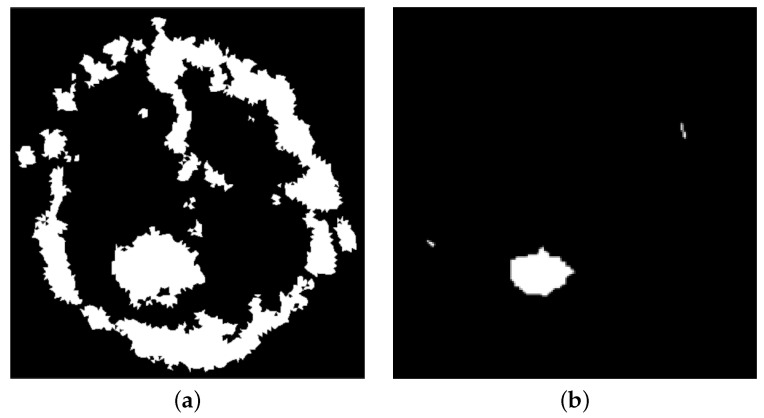
Binary map treatment. (**a**) obtained binary map *b* from definition (7); (**b**) post-processing result.

As the kernel slides through the image, a pixel is considered 1, and hence, white, if all the pixels under the kernel are 1; otherwise, it is eroded and its value is set to 0, i.e., black, see Figure 4. The erosion transformation is repeated up to 6 times per image. At the end of this phase, if the resulting image is completely black or if there are spurious white pixels accounting less than 1%, then the considered input MR I is automatically labelled as “healthy”; otherwise, it is automatically labelled as “affected”.

The proposed procedure is implemented in FreeFem ++, version 3.5; see [49] for the finite-elements approximation and is using the openCV (https://github.com/opencv/opencv-python (accessed on 30 August 2022)) Python library for the post-processing step. The tests are performed on a free dataset available on Kaggle (link for download https://www.kaggle.com/datasets/navoneel/brain-mri-images-for-brain-tumor-detection, accessed on 15 August 2022). There are in total 253 MR images of different sizes and of different modalities, i.e., T1, T2, but this information is not a priori provided. The affected brains are 155 while the healthy ones are 98. The images come split in two folders for the healthy and the affected ones so that a post evaluation of the performance of the algorithm is possible. In particular, sensitivity and miss rate are considered as indices for the evaluation. By using sensitivity, also called true positive rate, it is possible to see how many brain images, in the affected folder, are correctly labelled, as well as how many images are correctly classified as healthy, in the other folder. Note that the indicator sensitivity for the healthy brain class corresponds to computing the specificity indicator for the whole dataset. The indicator miss rate instead is giving insight into the wrongly classified images. By observing the results reported in Table 1, it is evident how the proposed strategy is more prone to identify anomalous regions, and hence, to label as “affected”, in most of the circumstances, any given input image. To understand better the reasons of this behavior, it is necessary to analyze the MR scans of healthy brains that are wrongly classified as affected, since this category is the one where the proposed algorithm is rather faulty, i.e., 63.27% of failure.

### 3.3. Correction

**Figure 5:**
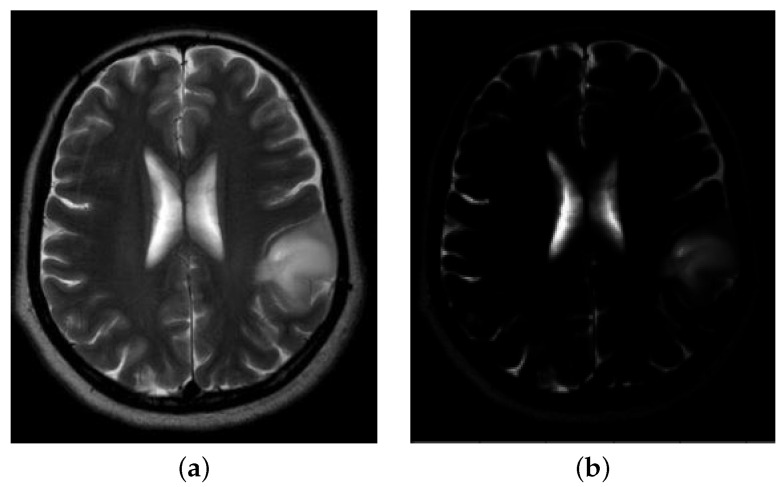
The cerebrospinal fluid appears with high intensity signal for T2-weighted MRIs. (**a**) image id N22: healthy brain; (**b**) image id N22 after gamma correction.

For certain images of healthy brains in the considered dataset, like the T2-weighted MR images, the cerebrospinal fluid appears with a high intensity signal [50], resulting therefore in bright areas, surrounded by dark pixels, that can be easily mistaken for anomalous sites after the gamma correction introduced in Section 3.1; see the example in Figure 5.

**Figure 6:**
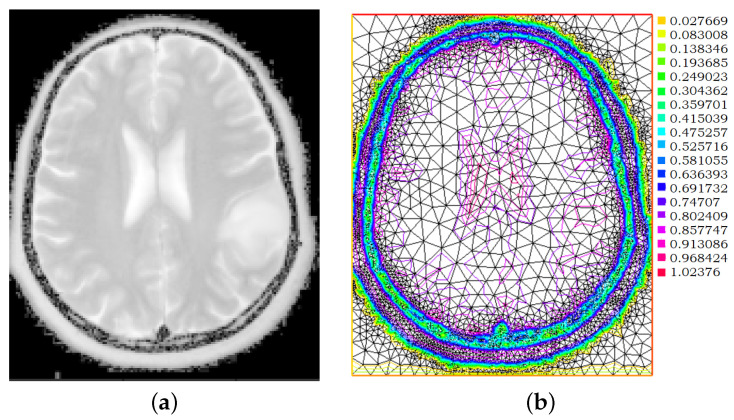

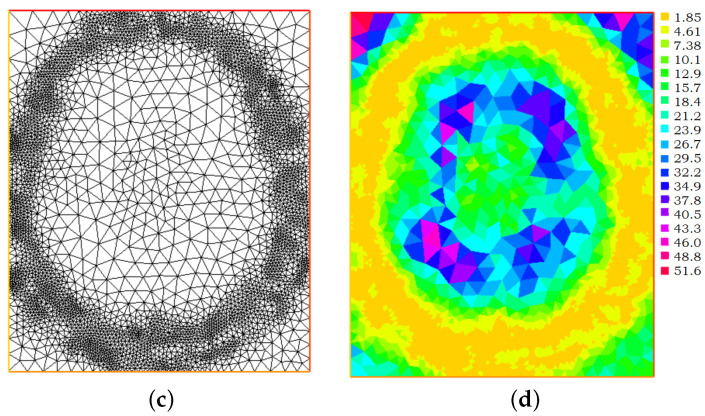
Treatment of the T2-weighted MR scans. (**a**) G for image id N22, γ=1/8; (**b**) isolines of the linear finite-elements approximation; (**c**) final resulting triangulation; (**d**) area of the triangles in the final triangulation.

Therefore, for such cases, the image G is produced by using γ=1/8. The results of this operation are shown in Figure 6. After this type of correction, the sensitivity index for the “healthy” category is increased to 72.45%, and the miss rate is reduced to 27.55%.

## 4. Discussion

The proposed procedure is globally evaluated by using two common metrics: the overall accuracy (*OA*) and the $F_{1}$ score. In particular, denoting with:

- *TP*: true positive, i.e., affected brains correctly classified;
- *TN*: true negative, i.e., healthy brains correctly classified;
- *FP*: false positive, i.e., healthy brains mistakenly classified as affected;
- *FN*: false negative, i.e., affected brains wrongly classified as healthy;

the overall accuracy is defined as

$$\begin{array}{c} OA:=\frac{TP+TN}{TP+TN+FP+FN}, \end{array}$$

while the $F_{1}$ score is computed as

$$\begin{array}{c} F_{1}:=\frac{2TP}{2TP+FP+FN}. \end{array}$$

The obtained results are displayed in Table 2 where also other competitors on the same dataset are shown. In particular, the performance of the considered competitors is published in [36] and regard the following methods: Auto-Encoder [32] and MemAE [51] are based on deep autoencoder architecture; AnoGAN [52], f-AnoGAN [53], GANomaly [54], Sparse-GAN [55], pix2pix [56], and Cycle-GAN [57] are based on generative adversarial networks; Proxy-Bridged [36] uses an intermediate proxy to bridge the input image and the reconstructed image to detect the anomalous regions.

The results in Table 2 clearly show the competitiveness of the proposed algorithm with respect to other methods; most of them even developed as supervised approaches in the tumor detection task.

## 5. Conclusions

In this paper, a method based on finite-elements and on the Z2 error estimator is presented to address the task of tumor detection in brain MR images. The proposed approach is called Z2-γ as it also relies on a suitable preliminary gamma correction which helps to better highlight the anomalous regions, whenever present. This initial study shows promising results in the application of the Z2 error estimator, together with isotropic triangulations, and specific tools of image processing in the automatic detection of brain tumors. Future work is currently devoted to provide fully automatic settings for the parameter γ, according to which input image is given, and to the study of brain–tumor segmentation in its various parts, i.e., active cells, necrotic core, and edema, see [58,59,60] and references therein, a task which requires a better discriminative power of the error estimator, as well as suitable input images; see [61]. Moreover, in the current approach, the final validation should always be carried out by the radiologist, as the method achieves 84.6% accuracy, and hence some MR images are still not correctly classified. In order to make the participation of the medical staff more active, for future upgrades, the segmentation task could be performed by combining both classical MRIs and functional-MRIs, and the initial screening of possible affected areas could be completed by the radiologist by drawing specific curves, as it is proposed in [62]. Although the resulting method would still not be fully automatic, the author believes that it is always important to rely on the supervision of competent medical staff as well as it is fundamental to develop suitable technologies to improve the accuracy and precision of any initial formulated diagnosis.

## Figures and Tables

**Table 1:** Sensitivity and miss rate for the affected and the healthy brains.

Index	Affected	Healthy
Sensitivity	92.26%	36.73%
Miss rate	7.74%	63.27%

**Table 2:** Competitors’ results on the same analyzed dataset. The best results are in bold.

Methods	OA	$F_{1}$
Z2-γ	**0.846**	**0.880**
Auto-Encoder	0.714	0.674
MemAE	0.789	0.722
AnoGAN	0.757	0.691
f-AnoGAN	0.764	0.675
GANomaly	0.798	0.667
Sparse-GAN	0.791	0.645
pix2pix	0.737	0.617
Cycle-GAN	0.752	0.712
Proxy-Bridged	0.805	0.709

## Data Availability

The dataset can be downloaded at: https://www.kaggle.com/datasets/navoneel/brain-mri-images-for-brain-tumor-detection (accessed on 30 August 2022).

## Abbreviations

The following abbreviations are used in this manuscript:

MRI	Magnetic Resonance Imaging
T1	Longitudinal relaxation time
T2	Transversal relaxation time
RT	Repetition time
RE	Time to echo
Z2	Zienkiewicz–Zhu error estimator
OA	Overall accuracy (or simply accuracy)
F_1	F_1 score
